# Autologous Chondrocyte Implantation Is Not Better Than Arthroscopic Debridement for the Treatment of Symptomatic Cartilage Lesions of the Knee: Two-Year Results From a Randomized-Controlled Trial

**DOI:** 10.1016/j.asmr.2024.100909

**Published:** 2024-02-16

**Authors:** Per-Henrik Randsborg, Jan E. Brinchmann, Christian Owesen, Lars Engebretsen, Thomas Birkenes, Heidi Andreassen Hanvold, Jūratė Šaltytė Benth, Asbjørn Årøen

**Affiliations:** a Department of Orthopaedic Surgery, Akershus University Hospital, Lørenskog, Norway; b Institute of Clinical Medicine, Faculty of Medicine, University of Oslo, Norway; c Department of Immunology, Oslo University Hospital, Rikshospitalet, Oslo, Norway; d Department of Orthopaedic Surgery, Haukeland University Hospital, Bergen, Norway; e Department of Physiotherapy, Akershus University Hospital, Lørenskog, Norway; f Health Services Research Unit, Akershus University Hospital, Lørenskog, Norway; g Oslo Sports Trauma Research Center, Norwegian School of Sport Sciences, Oslo, Norway; h Sports Traumatology and Arthroscopy Research Group, Bergen, Norway

## Abstract

**Purpose:**

To compare the functional and patient-reported outcome measures after autologous chondrocyte implantation (ACI) and arthroscopic debridement (AD) in symptomatic, isolated cartilage injuries larger than 2 cm^2^ in patients aged 18 to 50 years.

**Methods:**

Twenty-eight patients were included and randomized to ACI (n = 15) or AD (n = 13) and followed for 2 years. The primary outcome was the change in the Knee injury and Osteoarthritis Outcome Score (KOOS) Quality of Life (QoL) subscale.

**Results:**

The mean age at inclusion was 34.1 (standard deviation [SD] 8.5) years. There were 19 (68%) male patients. The mean size of the lesion was 4.2 (SD 1.7) cm^2^. There was a statistically significant and clinically meaningful improvement in patient-reported outcome measures from baseline to 2 years in both groups. The improvement from baseline to final follow-up for the primary endpoint (the KOOS QoL subscale) was larger for the AD group (39.8, SD 9.4) compared with the ACI group (23.8, SD 6.7), but this difference was not statistically significant (*P* = .17). However, according to a mixed linear model there were statistically significantly greater scores in the AD group for several KOOS subscales at several time points, including KOOS QoL, KOOS pain, and KOOS sport and recreation at 2 years.

**Conclusions:**

This study indicates that AD followed by supervised physiotherapy is equal to or better than ACI followed by supervised physiotherapy in patients with isolated cartilage lesions of the knee larger than 2 cm^2^. The improvement in KOOS QoL score from baseline to 2 years was clinically meaningful for both groups (23.8 points for ACI and 39.8 points AD), and larger for the AD group by 16 points.

**Level of Evidence:**

Level I, prospective randomized controlled trial.

Cartilage injuries of the knee are common in young, active adults and may lead to permanent damage, which in turn may cause severe functional impairment, pain, and swelling.^1^^,^^2^ Treatment of focal cartilage lesions of the knee is difficult, and there is no current gold standard for surgical management.^3^, ^4^, ^5^ The ideal treatment aims to recreate healthy normal hyaline cartilage tissue with the same durability as normal articular cartilage. Current treatment options include microfracture, mosaicplasty, autologous chondrocyte implantation (ACI), and osteochondral allograft.^3^, ^4^, ^5^, ^6^, ^7^, ^8^

ACI was introduced in the late 1980s in an attempt to reimplant the patient’s own chondrocytes, following in vitro expansion, into the cartilage lesion with the expectation that they would mature into hyaline-like cartilage.^9^^,^^10^ The purpose of arthroscopic debridement (AD) is to stabilize the lesion and to remove loose intra-articular fragments and inflammatory mediators down to the subchondral bone, but not through it.^11^

ACI has gained popularity, and several randomized controlled trials (RCTs) have compared ACI with other cartilage procedures, such as microfracture or mosaicplasty.^12^, ^13^, ^14^, ^15^, ^16^, ^17^ The results indicate improved knee function in carefully selected patients.^18^ However, the Cochrane database reported that there is insufficient evidence to conclude that ACI is superior to other methods.^5^

The purpose of this study is to compare the functional and patient-reported outcome measures (PROMs) after ACI and AD in symptomatic, isolated cartilage injuries larger than 2 cm^2^ in patients aged 18 to 50 years. We hypothesized that there would be no statistically or clinically meaningful difference between the groups 2 years after surgery.

## Methods

All patients provided written, informed consent before inclusion. The protocol was approved by the Regional Committee for Medical and Health Research Ethics North-Norway (approval number 2015/2200) and by the data protection officer of Akershus University Hospital. The study was registered in ClinicalTrials.com before inclusion of the first patient (NCT02636881). Patients referred to one of the collaborating institutions with a full-thickness or osteochondral defect in the weight-bearing area of the femoral condyles or trochlea larger than 2 cm^2^ were assessed for inclusion. The lesion had to be grade 3 or 4 according to the International Cartilage Regeneration & Joint Preservation Society classification,^19^^,^^20^ and symptomatic, with a Lysholm score of less than 75.^21^ The inclusion criteria were based on the recommendations by Brittberg et al.^9^ and include patients aged 18 to 50 years old with a stable knee, good range of motion, normal alignment (less than 5° varus or valgus measured on hip–knee–ankle angle images), and no sign of radiologically osteoarthritis according to the Kellgren–Lawrence classification.^22^ The weight-bearing, fixed-flexion posteroanterior radiographs were obtained with the SynaFlex positioning frame (Synarc Inc., Newark, CA).^23^

### Randomization

The (1:1) treatment allocation (ACI or AD) was decided by block randomization with blocks of size 6 and 8, using a computer generator (randomization.org). The allocation group was printed in faded text and concealed in opaque numerically marked envelopes. The printing and concealing were prepared by an assistant not involved in the study, to secure blinding.

### Surgical Procedure

All patients underwent a diagnostic arthroscopy, and patients randomized to AD had this procedure performed at the end. For patients randomized to ACI, the chondrocyte implantation took place 2 weeks later in a second-stage operation as described to follow. The ACI technique was based on the technique described by Brittberg et al.^9^

### Diagnostic Arthroscopy (All Patients)

Three standard incisions were made: supralateral to the patella and medial and lateral of the patellar tendon. A thorough systematic diagnostic arthroscopic examination was performed. Loose bodies were removed and any meniscal pathology addressed. Inflamed synovium was debrided. The focal cartilage lesion was then measured using a standard 4-mm arthroscopic probe and graded according to the International Cartilage Regeneration & Joint Preservation Society classification.

### ACI (Second Generation) (ACI Group Only)

ACI was a 2-stage surgical procedure involving harvesting of cartilage (stage 1) and implantation of the expanded chondrocytes 2 weeks later (stage 2). The surgeries took place at Akershus University Hospital. The harvested chondrocytes were expanded in vitro over a 2-week period between the 2 stages at the Ex Vivo Cell Laboratory at Oslo University Hospital. The procedures are detailed to follow:

#### Stage 1: Debridement and Cartilage Harvest

Stage 1 included a diagnostic arthroscopy with full inspection of the knee joint to ensure the inclusion criteria were fulfilled, as described previously. A cartilage biopsy was then obtained from the medial aspect of the femoral notch with a ring curette. If there was insufficient healthy cartilage in this area, the cartilage was harvested from the minimal weight-bearing aspect of the lateral femoral condyle.

#### In Vitro Chondrocyte Expansion

The cartilage biopsy was transported to the cell culture laboratory in a sterile tube containing 0.9 % NaCl. The biopsy was cut into tiny pieces, digested using collagenase, and expanded in vitro as described previously,^24^ with the exception that fibroblast growth factor 2 was added to the culture medium at 10 ng/mL. The chondrocytes were cultured for 2 weeks and resuspended in 0.9 % NaCl supplemented with 10% autologous serum to a treatment density of maximally 150 million cells/mL and a total volume maximally of 0.5 mL, before being transported back to Ahus on the day of implantation. The transport between the laboratory and the hospital takes approximately 20 minutes.

#### Stage 2: Implantation of Chondrocytes 2 Weeks After Initial Arthroscopy

The chondrocyte implantation was performed under general or spinal anaesthesia. A tourniquet inflated to 250 mm Hg was applied to the upper thigh to achieve a bloodless field. The lesion was accessed via a mini-open arthrotomy (medial or lateral depending on the location of the lesion) and curetted down to subchondral bone. The surrounding cartilage was debrided to healthy tissue, exposing the lesion to bare bone. Care was taken to avoid bleeding. A template of sterile aluminium foil was used to model the exact shape of the lesion, overcorrecting with 1 to 2 mm. The size of the lesion was carefully recorded. The aluminium template was used to cut out a matching piece of collagen sheet (Chondro-Gide; Geistlich Pharma, Wolhusen, Switzerland), which was used to contain the cells in the defect. The collagen sheet was sutured to the lesion with 6.0 resorbable stitches and sealed with fibrin glue, leaving an opening at the upper part for injection of the cells. The cells were slowly injected using a soft catheter before closing the final opening with a single suture and fibrin glue. The capsule was then closed with subcutaneous resorbable sutures, before closing the skin incision.

### Arthroscopic Debridement (AD Group Only)

After completing the diagnostic arthroscopy as described previously, the lesion was stabilized by debridement around the edges and down to the subchondral bone using a ring curette, but not through it. No specific cartilage treatment (such as microfracture) was performed. Intra-articular local anesthetics were not used due to the potential harmful effect on cartilage.^25^, ^26^, ^27^ All surgeons were experienced in the operative procedures before treating patients in the study.

### Rehabilitation

All patients were admitted to the hospital for 2 to 4 days. Both treatment groups underwent an identical rehabilitation previously described by Wondrasch et al.^28^ The active rehabilitation and education program consists of 3 phases; accommodation, rehabilitation, and return to activity (Table 1). The patients were seen by a physiotherapist on day 1 after surgery and instructed in range of motion exercises and restrictions according to phase 1. A local physiotherapist saw the patients within 2 weeks after discharge from the hospital, and guided the patients through phases 1-3. The rehabilitation consisted primarily of cardiovascular and knee/hip progressive resistance and neuromuscular training, including balance and plyometric exercises. The designated study physiotherapist directed the rehabilitation program in close communication with the patients’ local physiotherapists.

**Table 1:** Rehabilitation Protocol (Identical for Both Groups)

Rehabilitation Phases	Physiotherapy and Activities	Objectives	Criteria for Progression to Next Phase
Phase 1: Accommodation	Education/coaching Ice, elevation, and compression Isometric exercises Range of motion Gait training (no weight-bearing for 2 weeks)	Reduce pain and swelling Normalize range of motion Regain quadriceps control	No pain and swelling during activities of daily living (ADL) Flexion 90° Normalized quadriceps activity while walking (clinical evaluation by the physical therapist)
Phase 2: Rehabilitation	Stationary bike cycling Progressive knee and hip resistance training Neuromuscular training	Recovery of full range of motion Normalize muscle strength Dynamic joint stability during ADL	Full range of motion No pain or swelling during and after training sessions Equally distributed weight on the lower limbs during weight-bearing exercises with no shift of the trunk (visually assessed by the physical therapist) Ability to stand on 1 limb on a flat surface for at least 10 seconds
Phase 3: Return to activity	Knee and hip resistance training Neuromuscular training Cardiovascular training	Recovery of strength and neuromuscular control Return to activity/sport	Return to sport based on individual assessment

### Primary Endpoint

The Knee Injury and Osteoarthritis Outcome Score (KOOS) is a PROM validated for cartilage research studies.^29^ It assesses 5 domains: pain, symptoms, activity of daily living, sport and recreational function, and knee-related quality of life (QoL). The primary end-point was the difference in KOOS knee-related QoL subscale between the groups at 2-year follow-up.

### Secondary Endpoints

A combination of validated PROMs, self-explanatory questionnaires, and clinical parameters were the secondary endpoints:

• KOOS score: all subscales (except the knee-related QoL sub-scale, which is the primary endpoint);
• Tegner score; to evaluate the level of physical activity;
• Lysholm score; a condition-specific outcome score containing 8 domains; limp, locking, pain, stair-climbing, use of support, instability, swelling, and squatting; and
• Visual analog scale; a scale for pain, where 0 represents no pain and 10 represents the worst pain imaginable.

All outcome questionnaires were completed by the patients before surgery (baseline values) and at each scheduled follow-up appointments at 3, 6, 12, and 24 months.

### Statistical Analysis

A change in KOOS QoL of 8-10 has been shown to be clinically meaningful.^30^^,^^31^ Therefore, a difference between 2 treatment groups in change in KOOS QoL of 10 and standard deviation (SD) for change of 15 was assumed.^14^ With the power of 80% and significance level of 5%, the estimated minimum number of patients was 37 in each group to be able to demonstrate a significant difference by an independent samples *t*-test.

Demographic and clinical characteristics are presented as means and SDs, medians, and minimum–maximum values, or frequencies and percentages, as appropriate. The differences between the groups in baseline characteristics were tested by independent samples t-test, χ^2^ test, or median test with continuity correction, as appropriate. The difference between the groups in the primary endpoint (change in KOOS QoL from baseline to 2-year follow-up) was assessed by independent samples *t*-test, after ensuring that the distribution was parametric. Due to multiple measurement time points, repeated observations were available for each patient, and thus intrapatient correlations might be present. Therefore, to assess the differences between the groups in primary and secondary endpoints, linear mixed model with fixed effects for time dummy and group dummy and the interaction between these 2 was estimated. Random intercepts for patients were included. Post hoc analyses were performed to assess between-group comparisons at each time point. The results are presented as means and SDs with the groups at each time point and mean difference with corresponding 95% confidence intervals and *P* values derived from linear mixed model. Complications between the groups were compared by χ^2^ test. All tests were 2-sided. Results with *P* values less than .05 were considered statistically significant. The data analysis was conducted using STATA, version 17 (StataCorp, College Station, TX).

## Results

**Fig 1:**
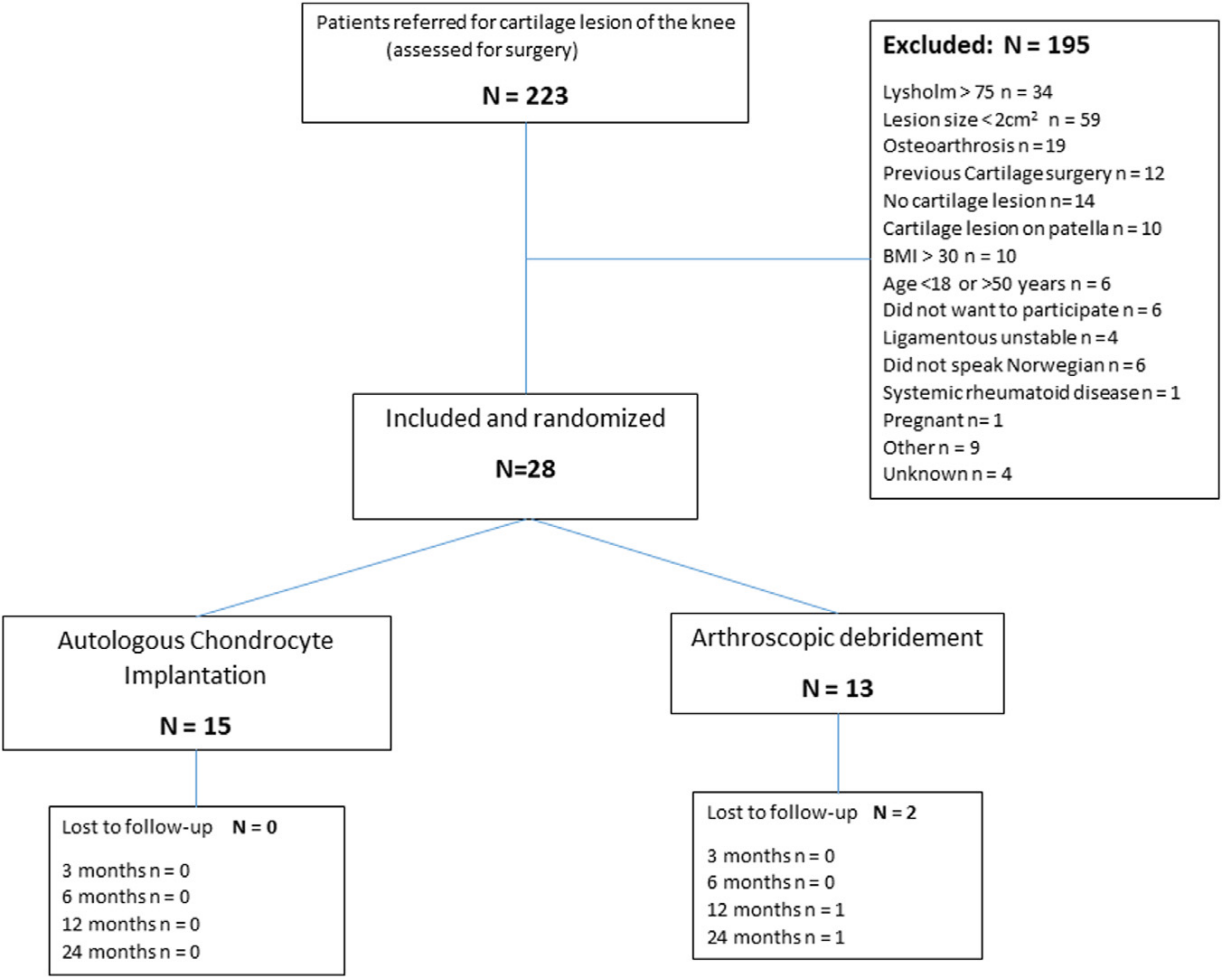
Flowchart of included patients. (BMI, body mass index.)

Between January 2016 and September 2022, 223 patients were referred to our study clinic, of which 28 patients were included and randomized (Fig 1). The mean age of the patients at the time of inclusion was 34.1 (ranging 20.5-48.2, SD 8.5) years. There were 19 (68%) men. The mean lesion size was 4.2 (ranging 2-9, SD 1.7) cm^2^. Most lesions were located on the medial femoral condyle (Table 2).

**Table 2:** Baseline Characteristics and Patient-Reported Outcomes in 28 Patients With a Symptomatic, Isolated, Full-Thickness Cartilage Lesion of the Knee, Randomized to Either ACI or AD

	Total (n = 28)	ACI (n = 15)	AD (n = 13)	*P* Value
Age, y, mean (SD)	34.1 (8.5)	33.5 (8.7)	34.9 (8.4)	.66∗
Males, n (%)	19 (68)	9 (60)	10 (77)	.40†
BMI, kg/m^2^ (SD)	25.8 (4.1)	26.5 (4.2)	25.1 (3.9)	.37∗
Lesion size, cm^2^, mean (SD)	4.2 (1.7)	4.0 (1.3)	4.5 (2.0)	
Right knee, n (%)	16 (57.1)	10 (66.7)	6 (46.2)	.27†
Operation time, min, mean (SD)	51.2 (25.7)	68.0 (21.6)	31.8 (13.5)	**<.001**∗
Anatomical location, n (%)				.44†
MFC	21 (75.0)	12 (80.0)	9 (69.2)	
LFC	4 (14.3)	1 (6.7)	3 (23.1)	
Trochlea	3 (10.7)	2 (13.3)	1 (7.7)	
Smokers, n (%)				.74†
Never	20 (71.4)	11 (73.3)	9 (69.2)	
Current smoker	3 (10.7)	1 (6.7)	2 (15.4)	
Previous	5 (17.9)	3 (20.0)	2 (15.4)	
Education, n (%)				.50†
Less than high school	3 (10.7)	1 (6.7)	2 (15.4)	
High school	13 (46.4)	8 (53.3)	5 (38.5)	
College	8 ((28.6)	3 (20.0)	5 (38.5)	
University (>4 y)	4 (14.3)	3 (20.0)	1 (7.7)	
Symptom duration, mo, median (min-max)	33.0 (2-180)	36.0 (8-180)	20.5 (2-60)	.11‡
PROMs, median (min-max)				
VAS pain sitting	2.6 (0.1-9.5)	2.3 (0.5-9.5)	3.0 (0.1-5.9)	.42‡
VAS pain standing	3.1 (0.5-9.5)	3.4 (0.5-9.5)	3.0 (0.5-8.5)	**.046**‡
Tegner Activity Scale	3 (0-7)	3 (0-6)	2 (0-7)	**.001**‡
Lysholm Score	54.5 (13-71)	54 (13-71)	55 (29-71)	.40‡
KOOS (symptoms)	46.4 (14.3-78.6)	46.4 (14.3-75.0)	50.0 (25.0-78.6)	.64‡
KOOS (pain)	54.2 (0-83.3)	52.8 (0-83,3)	58.3 (27.8-72.2)	.47‡
KOOS (ADL)	62.5 (13.2-94.1)	60.3 (13.2-92.6)	64.7 (27.9-94.1)	.20‡
KOOS (sport/rec)	22.5 (0-100)	15.0 (0-100)	30.0 (0-65)	**.02**‡
KOOS (QoL)	25.0 (0-50)	18.8 (0-50)	25.0 (12.5-37.5)	.29‡

NOTE. *P*-values in bold; statistically significant.

ACI, autologous chondrocyte implantation; AD, arthroscopic debridement; ADL, activities of daily living; BMI, body mass index; KOOS, Knee injury and Osteoarthritis Outcome Score; LFC, lateral femoral condyle; MFC, medial femoral condyle; PROMs, patient-reported outcome measures; QoL Quality of Life; rec, recreation; SD, standard deviation; VAS, visual analog scale.

∗ *t*-test.

† χ^2^ test.

‡ Continuity corrected median test.

### Patient-Reported Outcomes

**Fig 2:**
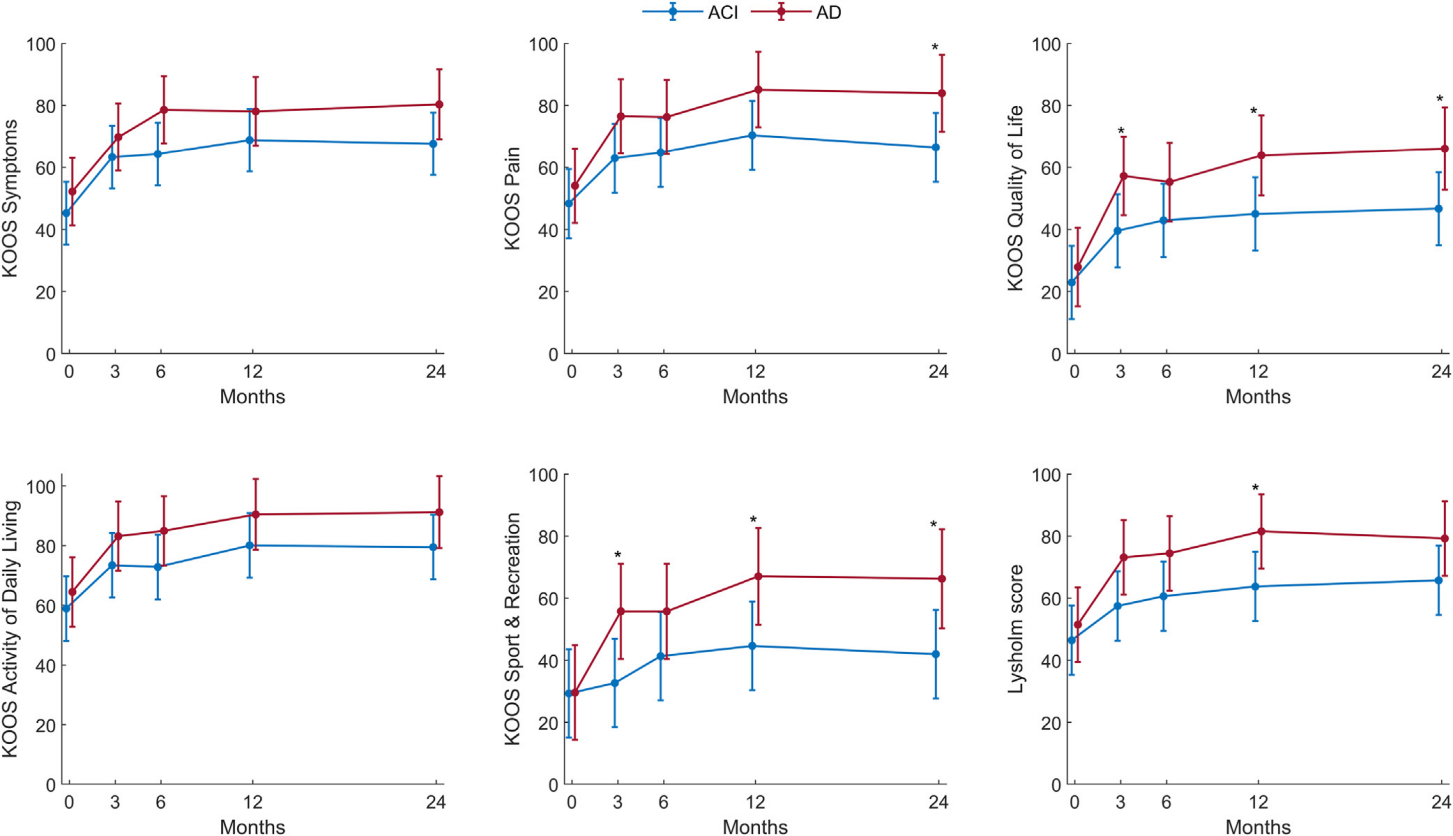
Comparison of patient-reported outcome between the groups during the 2-year follow-up period. The dots and I bars indicate the means and 95% confidence intervals, respectively, estimated by the linear mixed model. The asterisks indicate a statistically significant difference between groups at 5% significance level. (ACI, autologous chondrocyte implantation; AD, arthroscopic debridement; KOOS, Knee injury and Osteoarthritis Outcome Score.)

There was a statistically significant and clinically meaningful improvement in PROMs from baseline to 2-year follow-up in both groups, however, with no difference between the groups according to independent samples *t*-test (Table 3). However, a mixed linear model showed statistically significantly greater scores in the AD group for some KOOS subscales at several timepoints, including KOOS QoL, KOOS pain, and KOOS sport and recreation at 2 years (Table 4 and Fig 2).

**Table 3:** Primary Endpoint Analysis

Group	N	Mean Change in KOOS QoL From Baseline to 2-Year Follow-Up (SD)	Difference Between Groups in Mean Change	
			Mean Difference (95% CI)	*P* Value∗
ACI	15	23.8 (6.7)	–16.0 (–39.1 to 7.1)	.165
AD	11	39.8 (9.4)		

ACI, autologous chondrocyte implantation; AD, arthroscopic debridement; CI, confidence interval; KOOS, Knee injury and Osteoarthritis Outcome Score; QoL Quality of Life; SD, standard deviation.

∗ Independent-samples *t*-test.

**Table 4:** Patient-Reported Outcome in 28 Patients Randomized to Either ACI or AD of an Isolated Cartilage Lesion of the Knee Larger Than 2 cm^2^

Time	ACI		AD		AD vs ACI	*P* Value
	n	Mean (SD)	n	Mean (SD)	Mean Difference (95% CI)	
KOOS Quality of Life						
0	15	22.9 (17.1)	13	27.9 (7.5)	5.0 (–12.3 and 22.3)	.574
3	15	39.6 (25.3)	13	57.2 (18.0)	17.6 (0.3-34.9)	**.046**
6	15	42.9 (25.9)	13	55.3 (25.4)	12.4 (–4.9 and 29.7)	.161
12	15	45.0 (32.6)	12	63.5 (25.7)	18.9 (1.4-36.4)	**.035**
24	15	46.7 (25.9)	11	67.0 (29.2)	19.4 (1.6-37.2)	**.033**
KOOS symptoms						
0	15	45.2 (18.3)	13	52.2 (16.8)	7.0 (–7.9 and 21.8)	.357
3	15	63.3 (24.4)	13	69.8 (20.2)	6.5 (–8.4 and 21.3)	.394
6	15	64.3 (25.3)	13	78.6 (15.8)	14.3 (–0.5 and 29.1)	.059
12	15	68.8 (25.6)	12	78.3 (17.0)	9.3 (–5.7 and 24.3)	.224
24	15	67.6 (21.1)	11	82.5 (16.7)	12.7 (–2.5 and 27.9)	.101
KOOS pain						
0	15	48.3 (23.3)	13	54.1 (16.0)	5.7 (–10.6 and 22.1)	.491
3	15	63.0 (76.5)	13	76.5 (18.9)	13.5 (–2.8 and 29.9)	.105
6	15	64.8 (27.9)	13	76.3 (18.5)	11.5 (–4.9 and 27.8)	.169
12	15	70.4 (27.3)	12	85.9 (16.2)	14.8 (–1.7 and 31.2)	.080
24	15	66.5 (24.0)	11	86.9 (16.8)	17.5 (0.8-34.1)	**.040**
KOOS activity of daily living						
0	15	58.9 (24.2)	13	64.5 (20.5)	5.6 (–10.3 and 21.5)	.493
3	15	73.4 (27.9)	13	83.1 (19.7)	9.7 (–6.2 and 25.6)	.231
6	15	72.8 (31.4)	13	85.0 (16.7)	12.1 (–3.8 and 28.0)	.135
12	15	80.1 (23.8)	12	90.8 (12.1)	10.4 (–5.7 and 26.4)	.206
24	15	79.5 (21.5)	11	93.6 (9.8)	11.7 (–4.5 and 27.9)	.157
KOOS sport and recreation						
0	15	29.3 (29.2)	13	29.6 (23.8)	0.3 (–20.6 and 21.2)	.979
3	15	32.7 (29.8)	13	55.8 (30.3)	23.1 (2.2-44.0)	**.030**
6	15	41.3 (29.1)	13	55.8 (31.0)	14.4 (–6.5 and 35.3)	.176
12	15	44.7 (31.1)	12	69.2 (25.8)	22.4 (1.3-43.5)	**.038**
24	15	42.0 (29.2)	11	71.8 (28.8)	24.3 (2.9-45.6)	**.026**
VAS knee-pain standing						
0	15	4.6 (3.1)	13	3.7 (2.5)	–0.9 (–2.9 and 1.1)	.388
3	15	3.0 (3.2)	13	2.0 (2.2)	–1.0 (–3.0 and 1.0)	.342
6	15	3.2 (3.3)	13	2.1 (2.2)	–1.1 (–3.1 and 0.9)	.264
12	15	3.6 (3.2)	12	1.9 (2.1)	–1.5 (–3.5 and 0.5)	.137
24	15	3.2 (2.8)	11	1.6 (2.2)	–1.3 (–3.3 and 0.7)	.212
VAS knee-pain sitting						
0	15	3.5 (2.6)	13	2.6 (1.9)	–0.9 (–2.4 and 0.6)	.261
3	15	2.2 (2.8)	13	1.2 (1.4)	–1.2 (–2.7 and 0.3)	.182
6	15	1.7 (2.5)	13	1.3 (1.9)	–0.4 (–1.9 and 1.1)	.566
12	15	1.7 (2.4)	12	1.4 (1.5)	–0.3 (–1.9 and 1.2)	.661
24	15	1.6 (1.8)	11	0.8 (1.1)	–0.7 (–2.2 and 0.9)	.389
Tegner activity scale						
0	15	2.9 (1.9)	13	2.1 (2.0)	–0.9 (–2.1 and 0.4)	.167
3	15	1.9 (1.2)	13	2.9 (1.7)	1.1 (–0.2 and 2.3)	.088
6	15	2.8 (1.7)	13	3.8 (1.5)	1.0 (–0.3 and 2.2)	.118
12	15	2.5 (1.6)	12	3.6 (1.3)	1.0 (–0.3 and 2.2)	.118
24	15	2.8 (1.5)	11	4.0 (2.3)	1.0 (–0.3 and 2.2)	.141
Lysholm score						
0	15	46.5 (19.5)	13	51.5 (14.2)	5.0 (–11.4 and 21.4)	.550
3	15	57.5 (25.8)	13	73.2 (20.4)	15.7 (–0.7 and 32.1)	.061
6	15	60.6 (31.1)	13	74.5 (20.3)	15.9 (–2.5 and 30.3)	.097
12	15	63.7 (28.7)	12	81.6 (18.8)	17.8 (1.3-34.3)	**.035**
24	15	65.7 (22.5)	11	81.4 (20.0)	13.5 (–3.2 and 30.2)	.113

NOTE. The means and SDs are observed values at each time point, whereas the differences, Cis, and *P* values are estimated from a linear mixed model. *P*-values in bold; statistically significant.

ACI, autologous chondrocyte implantation; AD, arthroscopic debridement; CI, confidence interval; KOOS, Knee injury and Osteoarthritis Outcome Score; QoL Quality of Life; SD, standard deviation; VAS, visual analog scale.

### Complications

Major complications were defined as any reoperation of the knee, pulmonary embolism, and sepsis or deep (intra-articular) infection. Minor complications included any unscheduled return visits to the outpatient clinic or general physician, delayed wound closure, or transient pain and stiffness. There were no differences in major or minor complications between the groups (Table 5). Two patients in each treatment group were reoperated during the 2-year follow-up. One ACI-patient underwent debridement of scar tissue 19 months after the cell implantation. Another patient who underwent ACI suffered a pulmonary embolism, which resulted in inactivity. The same patient developed arthrofibrosis and underwent a manipulation under anesthesia and arthroscopic arthrolysis 6 months after the ACI. The patient continued to have chronic pain and stiffness, with a Lysholm score of 34 at the 2-year visit. One patient who received AD underwent meniscal surgery including decompression of a meniscal cyst 15 months after the index surgery. Another patient who received AD underwent a debridement of a cartilage flap 19 months after the index surgery. The minor complications consisted of wound issues and one incident of hemarthrosis that was aspirated 1 week after AD for diagnostic purposes and symptomatic relief.

**Table 5:** Overview of Complications in 28 Patients Treated for Focal Cartilage Lesion of the Knee With Either ACI or AD

Complications	ACI (N = 15)	AD (N = 13)	*P* Value∗
Major complications			
Reoperation (arthroscopy)	2	2	
Pulmonary embolism	1		
MUA (arthrofibrosis)	1		
Total major complications	4	2	.5
Minor complications			
Delayed wound closure	1	1	
Hemarthosis (aspirated)		1	
Total minor complications	1	2	.5
Total all complications	5	4	.9

NOTE. One patient suffered several complications.

ACI, autologous chondrocyte implantation; AD, arthroscopic debridement; MUA, manipulation under anesthesia.

∗ χ^2^ test.

## Discussion

There was no statistically significant difference between the groups in change in KOOS QoL from baseline to 2-year follow-up. However, the study demonstrated statistically significantly greater patient-reported KOOS scores at 2 years in patients treated with AD compared with ACI. It is interesting that we could not find any better results in the ACI group. The AD group did better during the follow-up period and reported statistically significantly greater KOOS scores at several time points, including the final follow-up. It is perhaps not surprising that the patient who received AD did better at early time points, as the AD procedure is much less invasive than the 2-stage ACI procedure. However, previous studies of ACI indicate that the results peak at 2 years,^32^, ^33^, ^34^, ^35^ suggesting that the negative effect of the more extensive surgery should have passed at this time point, while we found that the patients treated with AD still remained similar or better than the ACI group. There is a possibility that the implanted chondrocytes protect the knee better than the simple debridement in the long-run. However, a study by Knutsen et al.,^35^ the authors found no difference in patient reported outcome or risk of arthroplasty in patients randomized to either ACI or microfracture after 15 years of follow-up. Furthermore, cartilage surgery does not reduce the long-term risk of knee arthroplasty after a focal cartilage lesion, indicating that implanted chondrocytes do not reduce the risk of developing symptomatic osteoarthritis compared to less-invasive methods.^36^

A 3-stage ACI is a more invasive procedure than AD, yet there were no differences in the number or severity of complications between the groups, indicating that neither ACI nor AD are without risk. We did not observe complications or donor-site morbidity following arthroscopic harvesting of cartilage.^37^ However, it should be noted that our samples size is too small to detect possible real differences in rare, but serious complications, such as pulmonary embolism and arthrofibrosis.

The ACTIVE consortium recently published a large, multicenter RCT comparing ACI with alternative forms of cartilage management in patients who had previous failed cartilage surgery of the knee.^38^ The treatment method in the control group varied, with microfracture (49%) and mosaicplasty (28%) being the most common. Like us, they found no difference in Lysholm scores between ACI and less-invasive cartilage surgeries. The authors found that previous microfracture had a detrimental effect on the outcome of ACI. In our study, patients who had undergone previous cartilage surgery, such as microfracture, were excluded. Although different surgical treatments for cartilage lesions of the knee have been compared in several trials, no statistically significant differences have been found.^39^ This supports the suggestion that the improvement might be a result of the postoperative rehabilitation rather than the surgery itself.^12^^,^^28^^,^^40^ All clinical trials evaluating the outcome after cartilage surgery of the knee have put the patients through a strict, intensive, and prolonged postoperative rehabilitation. This will likely improve compliance to the rehabilitation and may partly explain the clinical improvement observed. We believe the individualized rehabilitation program in our study, supervised in close collaboration with the physiotherapist, ensured good compliance, and may explain the somewhat surprisingly good results in the AD group. Previous studies have indicated that active rehabilitation can delay or avoid the need for cartilage surgery in patients similar to ours. Wondrasch et al.^28^ implemented an active rehabilitation program in 48 patients scheduled for surgery of a focal cartilage lesion of the knee. After 3 months, there was a statistically significant and clinically meaningful improvement in KOOS score, load progression, and hop score, to the point where 31 (65%) of the patients declined surgery for their cartilage lesion. This indicates that short-term good results can be achieved with physiotherapy alone. Dozin et al.^12^ compared ACI versus mosaicplasty, where all candidates were evaluated arthroscopically with debridement of the lesion 6 months before definitive treatment. The patients completed an intensive rehabilitation protocol following the debridement, but before the cartilage surgery. Thirty-one percent of the candidates experienced substantial clinical improvement following the debridement and physiotherapy alone and needed no further surgical treatment, questioning cartilage surgery as first treatment choice. The authors conclude that further randomized clinical trials are needed, where ACI should be compared to debridement alone. The mean lesion size in our study was 4.2 cm^2^, which is quite large. Large lesions may be more amenable to an osteochondral allograft transplantation.^41^ However, a separate study would be needed to determine the effect of OATs compared with debridement and rehabilitation.

RCTs are a missing link in our knowledge of biological cartilage restoration and hampers further progression in cell therapy.^39^ This study is an attempt to fill this knowledge gap. The results indicate that the true effect of ACI is unknown, and larger multicenter RCTs with a control group not undergoing cartilage surgery is required to understand the potential benefit and limitations of advanced cell therapy in cartilage restoration surgery. There is a need for better tools to select the subgroup of patients who are more likely to benefit from ACI. ACI should not be performed outside of a clinical trial.

### Limitations

The main limitation is the small number of patients included. Under the same assumptions as for the original power calculation, the actual power, given our sample size of 28 patients, is approximately 40%. Low power increases the probability for committing a type II error, i.e., possibility for false-negative findings. The study had to be stopped due to slow inclusion and lack of funding. Strict inclusion criteria left us with few eligible patients. This is a well-known issue in cartilage research.^38^^,^^42^ The main reason for exclusion was previous cartilage surgery or too high body mass index.

In addition, the cartilage surgery was performed at a single institution, which may limit the external validity of the results. In the study, patients who underwent debridement were also admitted overnight to ensure the same instruction by physiotherapist and administration of a continuous passive motion machine. This might not reflect real-world practice, as arthroscopic procedures often are carried out as day surgery. Furthermore, 2 weeks of non–weight-bearing activity after ACI might be considered insufficient.

## Conclusions

This study indicates that AD followed by supervised physiotherapy is equal to or better than ACI followed by supervised physiotherapy in patients with isolated cartilage lesions of the knee larger than 2 cm^2^. The improvement in Disabilities of the Arm, Shoulder and Hand (DASH) score from baseline to 2 years was clinically meaningful for both groups (23.8 points for ACI and 39.8 points AD), and larger for the AD group by 16 points.

## Disclosure

The authors declare the following financial interests/personal relationships which may be considered as potential competing interests: The study was funded by the 10.13039/501100006095South-Eastern Norway Regional Health Authority (grant number 2015107). All authors (P-H.R., J.E.B., C.O., L.E., T.B., H.A.H., J.Š.B., A.Å.) declare that they have no known competing financial interests or personal relationships that could have appeared to influence the work reported in this paper. Full ICMJE author disclosure forms are available for this article online, as supplementary material.

## Supplementary Data

ICMJE author disclosure forms

ICMJE author disclosure forms

ICMJE author disclosure forms

ICMJE author disclosure forms

ICMJE author disclosure forms

ICMJE author disclosure forms

ICMJE author disclosure forms

ICMJE author disclosure forms
