# Supplementary material for: Autologous Chondrocyte Implantation Is Not Better Than Arthroscopic Debridement for the Treatment of Symptomatic Cartilage Lesions of the Knee: Two-Year Results From a Randomized-Controlled Trial
Source: Arthrosc Sports Med Rehabil. 2024 Feb 16;6(2):100909. doi: 10.1016/j.asmr.2024.100909 (PMC10943062; doi:10.1016/j.asmr.2024.100909)
Supplement: ICMJE author disclosure forms [file mmc1.docx]

**Declaration of interests**
 
☐ The authors declare that they have no known competing financial interests or personal relationships that could have appeared to influence the work reported in this paper.
 
☒ The authors declare the following financial interests/personal relationships which may be considered as potential competing interests:

| Asbjorn Aroen reports financial support was provided by South-Eastern Norway Regional Health Authority. If there are other authors, they declare that they have no known competing financial interests or personal relationships that could have appeared to influence the work reported in this paper. |
| --- |
