# Supplementary material for: Autologous Chondrocyte Implantation Is Not Better Than Arthroscopic Debridement for the Treatment of Symptomatic Cartilage Lesions of the Knee: Two-Year Results From a Randomized-Controlled Trial
Source: Arthrosc Sports Med Rehabil. 2024 Feb 16;6(2):100909. doi: 10.1016/j.asmr.2024.100909 (PMC10943062; doi:10.1016/j.asmr.2024.100909)
Supplement: ICMJE author disclosure forms [file mmc7.docx]

**Declaration of interests**
 
☐ The authors declare that they have no known competing financial interests or personal relationships that could have appeared to influence the work reported in this paper.
 
☒ The authors declare the following financial interests/personal relationships which may be considered as potential competing interests:

| no If there are other authors, they declare that they have no known competing financial interests or personal relationships that could have appeared to influence the work reported in this paper. |
| --- |
